# Supplementary material for: Social trauma engages lateral septum circuitry to occlude social reward
Source: Nature. 2022 Nov 30;613(7945):696–703. doi: 10.1038/s41586-022-05484-5 (PMC9876792; doi:10.1038/s41586-022-05484-5)
Supplement: Supplementary file 1 — Regions showing significant difference between SUS and CTRL males (iDISCO+ analysis). [file 41586_2022_5484_MOESM1_ESM.pdf]

nature portfolio

https://doi.org/10.1038/s41586-022-05484-5

Supplementary information

Social trauma engages lateral septum circuitry to occlude social reward

In the format provided by the authors and unedited

name	z score	p value	q value
Periventricular hypothalamic nucleus	7.30416698	2.19E-40	3.7381E-38
Periventricular zone	6.56956732	5.88E-31	5.0183E-29
Retrochiasmatic area	6.30863691	2.82E-10	1.6045E-08
Medial preoptic area	6.16932582	6.86E-10	2.9273E-08
Dentate gyrus	6.12249841	9.21E-10	3.1441E-08
Nucleus of the brachium of the inferior colliculus	5.88422715	4.00E-09	1.1379E-07
Lateral amygdalar nucleus	5.61413035	1.98E-08	4.8281E-07
Temporal association areas	5.50286926	3.74E-08	7.9798E-07
Ectorhinal area	5.33940134	9.33E-08	0.00000177
Posterior auditory area	5.304406	1.13E-07	0.00000193
Peripeduncular nucleus	5.07434583	3.89E-07	0.00000553
Nucleus sagulum	5.07436943	3.89E-07	0.00000553
Periaqueductal gray	4.88711768	1.02E-06	0.00001339
Anterior hypothalamic nucleus	4.71763472	2.39E-06	0.00002914
Lateral preoptic area	4.6498689	3.32E-06	0.00003778
Parastrial nucleus	4.54346467	5.53E-06	0.00005899
Subiculum	4.47445819	7.66E-06	0.00007691
Midbrain	4.36168334	1.29E-05	0.00012233
Anterior cingulate area	4.23540028	2.28E-05	0.00019885
Endopiriform nucleus	4.23074881	2.33E-05	0.00019885
Primary auditory area	4.21928602	2.45E-05	0.00019914
Anterodorsal preoptic nucleus	4.16862786	3.06E-05	0.00023741
Midbrain reticular nucleus	4.05727492	4.96E-05	0.0003681
Mediodorsal nucleus of thalamus	4.04186663	5.30E-05	0.00037694
Ventromedial hypothalamic nucleus	4.03136856	5.55E-05	0.00037893
Anterior cingulate area	3.97707136	6.98E-05	0.00044948
Bed nucleus of the accessory olfactory tract	3.9726628	7.11E-05	0.00044948
Medial habenula	3.95980236	7.50E-05	0.00045721
Medial geniculate complex	3.94138094	8.10E-05	0.00047675
Thalamus	3.89439673	9.84E-05	0.00055986
Precommissural nucleus	3.8858349	0.00010198	0.00056152
Ventral auditory area	3.81315878	0.0001372	0.00073183
Medial preoptic nucleus	3.80171069	0.0001437	0.00074328
Paraventricular hypothalamic nucleus	3.78200563	0.00015557	0.00078101
Hippocampal formation	3.76856181	0.00016419	0.00080073
Posterior limiting nucleus of the thalamus	3.75408098	0.00017398	0.00082491
Parafascicular nucleus	3.74580652	0.00017982	0.00082955
Tuberomammillary nucleus	3.67377479	0.00023899	0.00107351
Anteroventral periventricular nucleus	3.61058746	0.0003055	0.00127185
Substantia nigra	3.62011853	0.00029447	0.00127185
Pedunculopontine nucleus	3.61387792	0.00030165	0.00127185
Interanterodorsal nucleus of the thalamus	3.57022944	0.00035667	0.00144952
Field CA3	3.55910003	0.00037213	0.00147718
Hypothalamus	3.5404474	0.00039945	0.00154959
Nucleus of the posterior commissure	3.52982439	0.00041584	0.00157733
Primary motor area	3.52364033	0.00042566	0.00157948
Perirhinal area	3.51511711	0.00043956	0.00159635
Paraventricular nucleus of the thalamus	3.48634664	0.00048967	0.00170575
Cuneiform nucleus	3.49008017	0.00048288	0.00170575
Cortical subplate	3.46135955	0.00053745	0.00183475
Anteroventral preoptic nucleus	3.42675058	0.00061085	0.00204443
Secondary motor area	3.41700179	0.00063315	0.00206329
Superior colliculus	3.41378799	0.00064066	0.00206329
Field CA1	3.37124744	0.00074829	0.00232228
Subparafascicular nucleus	3.37221355	0.00074567	0.00232228
Lateral hypothalamic area	3.3655509	0.00076391	0.00232842
Medial pretectal area	3.33729534	0.00084598	0.00253334
Nucleus of the trapezoid body	3.31593294	0.00091338	0.00268801
Entorhinal area	3.29154304	0.00099639	0.00283456
Subceruleus nucleus	3.29363969	0.00098899	0.00283456
Arcuate hypothalamic nucleus	3.27984313	0.00103865	0.00290635
Olfactory areas	3.2486724	0.00115945	0.00319204
Inferior colliculus	3.19161684	0.00141479	0.00383318
Substantia nigra	3.16668773	0.00154186	0.00411219
Cortical amygdalar area	3.15427501	0.00160897	0.00422516
Periventricular hypothalamic nucleus	3.13242437	0.00173369	0.00448369
Entorhinal area	3.07935526	0.00207449	0.005285
Subthalamic nucleus	3.0453502	0.0023241	0.00583383
Ventrolateral preoptic nucleus	3.02938362	0.00245053	0.00606204
Dorsomedial nucleus of the hypothalamus	3.01840713	0.00254107	0.00619622
Primary somatosensory area	3.00297101	0.00267358	0.00642751
Parasubthalamic nucleus	2.99568067	0.00273833	0.00649174
Cortical amygdalar area	2.99001762	0.00278961	0.00652272
Retrosplenial area	2.94874572	0.00319066	0.00724433
Gigantocellular reticular nucleus	2.94538346	0.00322555	0.00724433
Magnocellular reticular nucleus	2.95075161	0.00317002	0.00724433
Preparasubthalamic nucleus	2.93000364	0.00338958	0.00741753
Pons	2.93290874	0.00335803	0.00741753
Tuberal nucleus	2.91752118	0.00352826	0.00752798
Midbrain reticular nucleus	2.92043429	0.00349544	0.00752798
Primary somatosensory area	2.90454519	0.00367787	0.00768135
Magnocellular nucleus	2.89223229	0.00382515	0.00768135
Mammillary body	2.90070279	0.00372327	0.00768135
Ventral premammillary nucleus	2.89276396	0.00381868	0.00768135
Pons	2.89535713	0.00378727	0.00768135
Parasubiculum	2.85324771	0.00432749	0.00849034
Lateral habenula	2.85563037	0.00429515	0.00849034
Ventral tegmental area	2.84684662	0.00441546	0.00856449
Presubiculum	2.83563276	0.0045735	0.00877136
Primary somatosensory area	2.80860494	0.00497567	0.00943663
Vascular organ of the lamina terminalis	2.79022642	0.00526712	0.00966715
Parabrachial nucleus	2.7920469	0.00523758	0.00966715
Medulla	2.79332351	0.00521695	0.00966715
Piriform-amygdalar area	2.78417128	0.00536647	0.00974471
Anterior amygdalar area	2.77070389	0.00559353	0.01000803
Parapyramidal nucleus	2.7686592	0.00562875	0.01000803
Nucleus of Darkschewitsch	2.76240574	0.00573771	0.0100966
Nucleus x	2.73873763	0.00616756	0.01074225
Anterodorsal nucleus	2.71473542	0.00663287	0.01143601
Basolateral amygdalar nucleus	2.69592724	0.0070193	0.01198124
Dorsal auditory area	2.68363248	0.00728271	0.01230778
Nucleus of the lateral olfactory tract	2.66691064	0.0076552	0.01281045
Medial amygdalar nucleus	2.64622072	0.00813967	0.01348893
Parvicellular reticular nucleus	2.63600134	0.00838894	0.01376835
Primary somatosensory area	2.6312774	0.00850646	0.01382826
Supraoptic nucleus	2.62106699	0.0087655	0.01411494
Subparaventricular zone	2.6163981	0.00888629	0.01417571
Retrosplenial area	2.61316729	0.00897074	0.01417792
Striatum-like amygdalar nuclei	2.57044318	0.01015685	0.01590525
Piriform area	2.54291161	0.0109933	0.01696122
Postpiriform transition area	2.53861324	0.01112928	0.01696122
Vestibular nuclei	2.5397312	0.01109377	0.01696122
Dorsal part of the lateral geniculate complex	2.51771004	0.01181205	0.01784247
Posterior amygdalar nucleus	2.50709542	0.01217278	0.01822607
Retrosplenial area	2.47491006	0.01332697	0.01959576
Lateral septal complex	2.46891218	0.01355245	0.01959576
Posterior complex of the thalamus	2.46726946	0.01361479	0.01959576
Supramammillary nucleus	2.47538987	0.01330908	0.01959576
Dorsal premammillary nucleus	2.46604067	0.01366158	0.01959576
Lateral dorsal nucleus of thalamus	2.45248945	0.01418715	0.02018004
Median preoptic nucleus	2.44544448	0.01446737	0.02040856
Central amygdalar nucleus	2.4167386	0.01566026	0.02191024
Lateral posterior nucleus of the thalamus	2.40310396	0.01625657	0.02255963
Postsubiculum	2.39111774	0.01679717	0.02303796
Globus pallidus	2.3895028	0.0168712	0.02303796
Substantia innominata	2.35844963	0.01835145	0.02486039
Prelimbic area	2.32824055	0.01989933	0.02674501
Olfactory tubercle	2.29067345	0.02198231	0.02931375
Fasciola cinerea	2.28449942	0.0223422	0.02956271
Field CA2	2.25964941	0.02384302	0.03123791
Claustrum	2.25754153	0.02397426	0.03123791
Bed nuclei of the stria terminalis	2.23715498	0.02527621	0.03268482
Supplemental somatosensory area	2.22053074	0.02638276	0.0338592
Paragigantocellular reticular nucleus	2.20532694	0.02743116	0.03494198
Nucleus of reunions	2.2000584	0.02780275	0.03515297
Primary somatosensory area	2.19179488	0.02839432	0.03563696
Posterolateral visual area	2.16558682	0.03034277	0.03780443
Pallidum	2.1287056	0.03327863	0.04116181
Striatum	2.11744112	0.03422243	0.04194068
Posterior hypothalamic nucleus	2.11535434	0.03439976	0.04194068
Diagonal band nucleus	2.11137948	0.03473971	0.04205476
Superior olivary complex	2.09045704	0.03657676	0.04396681
Primary somatosensory area	2.07582213	0.0379104	0.04525123
Anterior pretectal nucleus	2.06973861	0.03847683	0.0456084
Nucleus accumbens	2.06581226	0.03884621	0.04572869
Anteromedial visual area	2.06146795	0.03925842	0.0458974
Oculomotor nucleus	2.04912648	0.04044975	0.04696849
Sublaterodorsal nucleus	2.02802876	0.04255731	0.04908181
