# Supplementary material for: Social trauma engages lateral septum circuitry to occlude social reward
Source: Nature. 2022 Nov 30;613(7945):696–703. doi: 10.1038/s41586-022-05484-5 (PMC9876792; doi:10.1038/s41586-022-05484-5)
Supplement: Supplementary file 2 — Reporting Summary [file 41586_2022_5484_MOESM2_ESM.pdf]

nature portfolio

Corresponding author(s): Scott J. Russo

Last updated by author(s): Sep 22, 2022

Reporting Summary

Nature Portfolio wishes to improve the reproducibility of the work that we publish. This form provides structure for consistency and transparency in reporting. For further information on Nature Portfolio policies, see our Editorial Policies and the Editorial Policy Checklist.

Statistics

For all statistical analyses, confirm that the following items are present in the figure legend, table legend, main text, or Methods section.

n/a	Confirmed	
☐	☒	The exact sample size (*n*) for each experimental group/condition, given as a discrete number and unit of measurement
☐	☒	A statement on whether measurements were taken from distinct samples or whether the same sample was measured repeatedly
☐	☒	The statistical test(s) used AND whether they are one- or two-sided<br>*Only common tests should be described solely by name; describe more complex techniques in the Methods section.*
☒	☐	A description of all covariates tested
☐	☒	A description of any assumptions or corrections, such as tests of normality and adjustment for multiple comparisons
☐	☒	A full description of the statistical parameters including central tendency (e.g. means) or other basic estimates (e.g. regression coefficient) AND variation (e.g. standard deviation) or associated estimates of uncertainty (e.g. confidence intervals)
☐	☒	For null hypothesis testing, the test statistic (e.g. *F*, *t*, *r*) with confidence intervals, effect sizes, degrees of freedom and *P* value noted<br>*Give P values as exact values whenever suitable.*
☒	☐	For Bayesian analysis, information on the choice of priors and Markov chain Monte Carlo settings
☒	☐	For hierarchical and complex designs, identification of the appropriate level for tests and full reporting of outcomes
☐	☒	Estimates of effect sizes (e.g. Cohen's *d*, Pearson's *r*), indicating how they were calculated

Our web collection on statistics for biologists contains articles on many of the points above.

Software and code

Policy information about availability of computer code

Data collection	Social CPP data was acquired using CPP box(Med Associates). Other behaviors were assayed using Ethovision XT 11/12 (Noldus). Electrophysiological data was performed with pClamp 10.0 (Molecular Devices). Fiber photometry data was acquired with open source Bonsai software 2.4.0 and custom Neurophotometrics (Neurophotometrics, Ltd) hardware. Cleared brain imaging was done on lightsheet microscope (LaVision).
Data analysis	Analysis of electrophysiological data was performed with Clampfit 10.0 (Molecular Devices). Analysis of fiber photometry data was performed with Matlab 2019/2020/2021 (Mathworks, Inc.). Histological data was analyzed using ImageJ1.52b(Fiji). Cleared brain data was analyzed by ClearMap (python2.7). Statistics are done by using GraphPad Prism8 and 9. All MATLAB code for calcium imaging process, Python code for iDISCO+ analysis can be obtained from github (https://github.com/nyclong/2021-07-11642-Nature.git)

For manuscripts utilizing custom algorithms or software that are central to the research but not yet described in published literature, software must be made available to editors and reviewers. We strongly encourage code deposition in a community repository (e.g. GitHub). See the Nature Portfolio guidelines for submitting code & software for further information.

Data

Policy information about availability of data

All manuscripts must include a data availability statement. This statement should provide the following information, where applicable:

- Accession codes, unique identifiers, or web links for publicly available datasets
- A description of any restrictions on data availability
- For clinical datasets or third party data, please ensure that the statement adheres to our policy

All raw data for animal behaviors, ISH and IHC statistics are available as source data files. Allen Brain Atlas ISH database is used to search for possible molecular markers in lateral septum.

Field-specific reporting

Please select the one below that is the best fit for your research. If you are not sure, read the appropriate sections before making your selection.

☒ Life sciences ☐ Behavioural & social sciences ☐ Ecological, evolutionary & environmental sciences

For a reference copy of the document with all sections, see nature.com/documents/nr-reporting-summary-flat.pdf

Life sciences study design

All studies must disclose on these points even when the disclosure is negative.

Sample size	Sample sizes were chosen according to previous experiments (Chaudhury et al. 2013, Friedman et al. 2014, Christoffel et al. 2015)
Data exclusions	Grubb's test were used to exclude significant outliers. This test was only performed once per data set. In experiments requiring viral infection of a specific brain region, mice were excluded from behavioral analysis if the virus was found to be mis-targeted or not expressed according to pre-determined anatomical criteria. No data was excluded for other reasons.
Replication	Data was collected using biological replicates (e.g. multiple brain slices per animal analyzed for in-situ hybridization and Immunohistochemistry). All attempts at replication were successful. Fig. 2c and Extended Data Fig. 3i were repeated in 3 separate cohorts per sex, with all showing similar results. Fig. 5a, b (right panel) was repeated in 3 seperate male cohorts (n = 6) and 1 female cohort (n = 2) with all showing similar results. Extended Data Fig. 3j was repeated twice in both sexes, with both showing similar results. Extended Data Fig. 6a was repeated in 4 separate cohorts in both sexes with all showing similar results. Extended Data Fig. 8a, d (right panel) and 10e were repeated twice in males only, with both cohorts showing similar results. Extended Data Fig. 8b was repeated three times, with all showing similar results.
Randomization	Animals and samples were assigned randomly to control and experimental groups, except in cases where social behaviors were compared between groups. In these cases, SI ratio was assessed and groups were counter-balanced for equal levels of SI ratio before manipulations were performed.
Blinding	Experimenters were blind to group allocation except for social CPP data collection since we need to pre-test social CPP and assign boxes for different groups of animals (CTRL or RES or SUS) separately, we need to know which ones belong to which groups so that we can balance the conditioned chambers to avoid bias, analyses were performed blind to experimental conditions<br>(Behavioral scoring from videos, fiber photometry analysis, quantification of in-situ hybridization results).

Reporting for specific materials, systems and methods

We require information from authors about some types of materials, experimental systems and methods used in many studies. Here, indicate whether each material, system or method listed is relevant to your study. If you are not sure if a list item applies to your research, read the appropriate section before selecting a response.

Materials & experimental systems

n/a	Involved in the study	
☐	☒	Antibodies
☒	☐	Eukaryotic cell lines
☒	☐	Palaeontology and archaeology
☐	☒	Animals and other organisms
☒	☐	Human research participants
☒	☐	Clinical data
☒	☐	Dual use research of concern

Methods

n/a	Involved in the study	
☒	☐	ChIP-seq
☒	☐	Flow cytometry
☒	☐	MRI-based neuroimaging

Antibodies

Antibodies used	Mouse monoclonal IgG anti-c-Fos (Santa Cruz Biotechnology, C-10, Cat. No.: sc-271243, 1:1000)<br>Rabbit Polyclonal anti-cfos antibody (synaptic systems, Cat. No.: 226 003, 1:1000)<br>Cy™2 AffiniPure Donkey Anti-Rabbit IgG (H+L), Jackson ImmunoResearch Laboratories, Inc.Code Number: 711-225-152, Lot Number: 78325, Clonality: Polyclonal, RRID: AB_2340612, 1:1000;<br>Cy™3 AffiniPure Donkey Anti-Rabbit IgG (H+L), Jackson ImmunoResearch Laboratories, Inc.Code Number: 711-165-152, Lot Number: 88067, Clonality: Polyclonal, RRID: AB_2307443, 1:1000;<br>Cy™5 AffiniPure Donkey Anti-Rabbit IgG (H+L), Jackson ImmunoResearch Laboratories, Inc.Code Number: 711-175-152, Lot Number: 84963, Clonality: Polyclonal, RRID: AB_2340607, 1:1000.<br>Donkey anti-Rabbit IgG (H+L) Highly Cross-Adsorbed Secondary Antibody, Alexa Fluor™ 647, Thermo Fisher Scientific, cat: A-31573, RRID, AB_2536183, 1:1000
Validation	According to manufacturers, mouse polyclonal IgG anti-cFos was validated in mouse and human tissue for immunofluorescence. This antibody has been utilized in previous publications to detect cfos in mice using immunofluorescence (Liu, J. et al.,2017).<br>According to manufacturers, Rabbit Polyclonal anti-cfos antibody IgG was validated in transgenic mice expressing GFP using immunofluorescence. This antibody has been used in previous publications to detect cfos in mice using immunofluorescence (Li, X, et al., 2021).<br>According to manufacturers, Cy™2/3/5 AffiniPure Donkey Anti-Rabbit IgG (H+L) was validated in mice. This antibody has been used in previous publications in mice using immunofluorescence (HE, X., et al., 2018).<br>According to manufacturers, Donkey anti-Rabbit IgG (H+L) Highly Cross-Adsorbed Secondary Antibody, Alexa Fluor™ 647 was validated in mice. This antibody has been used in previous publications in mice using immunofluorescence (Kyprianou, C., et al., 2020).

Animals and other organisms

Policy information about studies involving animals; ARRIVE guidelines recommended for reporting animal research

Laboratory animals	C57BL6/J: Obtained from Jackson Laboratory (male and female), 4-10 weeks of age, cat. 000664).<br>NT-Cre (017525-B6;129-Nts <tm1(cre) Mgmj>/J; Jackson Laboratory, Stock No: 017525), (male and female) 6-10 weeks of age. Heterozygous mice were used for all experiments.<br>CD1: Obtained from Charles River labs (male, 4-6 months of age, cat. CRL22). Male mice were used as aggressors and not studied here.<br>ERα-Cre: B6N.129S6(Cg)-Esr1tm1.1(cre)And/J, Obtained from Jackson Laboratory (male, 10 weeks of age, Stock No: 017911), were crossed with CD1 wild-type mice for one generation to generate ERα-Cre/CD1 F1 hybrids. Male F1 hybrids 4-6 months of age were used for experiments. Female F1 hybrids were co-housed with F1 males prior to testing. These mice were used as aggressors and not studied here.
Wild animals	No wild animals were used in the study.
Field-collected samples	No field collected samples were used in the study.
Ethics oversight	Procedures were performed in accordance with the National Institutes of Health Guide for Care and approved by the Use of Laboratory Animals and the Icahn School of Medicine at Mount Sinai Institutional Animal Care and Use Committee. Additional information about mice used in this study can be found in the Life Sciences Reporting Summary.

Note that full information on the approval of the study protocol must also be provided in the manuscript.
